# The Childbirth Experiences of Pregnant Women Living with HIV Virus: Scoping Review

**DOI:** 10.3390/children11060743

**Published:** 2024-06-18

**Authors:** Andréa Paula de Azevedo, Jorge Fonte de Rezende Filho, Cristina Barroso Hofer, Francisca Rego

**Affiliations:** 1 Faculty of Medicine, University of Porto, 4200-319 Porto, Portugal; 2 Institute of Childcare and Pediatrics Martagão Gesteira, Federal University of Rio de Janeiro, Rua Bruno Lobo, 50, Rio de Janeiro 21941-912, Brazil; 3 National Medicine Academy, Rio de Janeiro 20021-130, Brazil; 4 Maternity School, Federal University of Rio de Janeiro, Rio de Janeiro 22240-001, Brazil; 5 Department Infectious Diseases, School of Medicine, Federal University of Rio de Janeiro, Rio de Janeiro 21044-020, Brazil

**Keywords:** childbirth, experience, pregnant women, HIV, scoping review

## Abstract

OBJECTIVE: Understand and explore the childbirth experiences of pregnant women living with HIV (PWLWHIV). With the advent of several measures to decrease the intrapartum HIV infection and a strong emphasis on the humanization of childbirth, there is a growing focus on providing positive childbirth experiences for pregnant women. Indeed, a positive childbirth experience is even more important in the group of pregnant women living with HIV (PWLWHIV) as it plays a pivotal role in enhancing the mother’s adherence to her postpartum treatment and the newborn’s engagement in Infectious Disease services. METHODOLOGY: A scoping review was conducted. Searches were performed on databases, such as MEDLINE, PUBMED, WEB OF SCIENCE and Cochrane Library, using the following keywords: childbirth, birth, parturition, HIV, humaniz*, perceived safety, experience, maternal satisfaction, healthcare professional and midwi*. Articles meeting pre-established criteria were selected within the timeframe of 2013 to 2023 for inclusion in the review. RESULTS: Out of a total of 2,340,391 articles, 4 were chosen based on our defined criteria. Three primary themes emerged from the selected articles: the assessment of childbirth experience quality, vulnerability and autonomy. CONCLUSIONS: The four studies identified had a small sample size and were not adequately conducted with a specific focus on studying the childbirth experience of pregnant women living with HIV (PWLWHIV). This scoping review revealed a gap in the existing literature, indicating a need for further research and clarification in the identified area.

## 1. Introduction

In accordance with the Sustainable Development Goals (SDGs) outlined by the United Nations, the Global Strategy for Women’s, Children’s, and Adolescents’ Health (2016–2030) represents a pivotal change in the emphasis placed on public health priorities [1,2]. It is necessary to improve the care of pregnant woman living with HIV (PWLWHIV), not only in reducing the HIV maternal-to-child vertical transmission (MTCT) but also improving the PWLWHIV childbirth experience, to enhance their commitment to treatment during the postpartum phase and to facilitate follow-up care for the newborn. This approach aims to decrease morbidity and mortality among pregnant women living with HIV (PWLWHIV) and lower the incidence of new HIV cases in newborns. Ensuring a positive childbirth experience aligns with one of the goals set by UNICEF [3].

Since the early 2000s, women have accounted for approximately 25% of positive HIV test results in Canada. Women of reproductive age constitute the fastest-growing demographic affected by HIV infection in Canada [4]. This percentage is increasing due to the successful combination of antiretroviral therapy with low viral loads and the Prevention of Mother-to-Child Transmission Program (PMTCT) [5], which has a transmission rate of less than 1% [6]. The PMTCT program aims to reduce vertical and sexual transmission of HIV and improve the postpartum retention of WLWHIV [7].

Globally, the rate of PWLWHIV increased from 1.5/1000 live births in 2010 to 3.3/1000 live births in 2021 [8]. HIV infection greatly impacts the quality of life of pregnant women and has a significant impact on childbirth and the postpartum period [9]. Without diagnosis and without prenatal and intrapartum care, the vertical transmission rate is around 28% [6]. With prenatal and perinatal care (antiretroviral treatment, undetectable viral load prior to the delivery, no amniotomy or with a duration of less than 4 h), the vertical transmission rate is reduced to less than 1–2% [4,9].

The satisfaction of the pregnant woman is an indicator of quality care, which is becoming increasingly important [10]. The concept of birth satisfaction is important for women, healthcare professionals, healthcare administrators and policymakers [11]. The satisfaction of women during childbirth has immediate and long-term effects on their health, the health of their children, family relationships, healthcare policies, breastfeeding and even population growth [12]. Using a definition from the literature, the childbirth experiences encompass the sensations and long-term memories of the pregnant woman, including emotions, a sense of well-executed work, competence, confidence and decision-making power [13]. These experiences are complex, multidimensional and subjective. It is generally based on the outcomes of the experiences, pre-existing expectations or desires and the differences between what was expected or desired and what was actually experienced [14]. The pregnant woman’s perception of safety is identified as one of the key factors influencing childbirth satisfaction [15].

PWLWHIV have specific needs during the perinatal period because they require specific knowledge about the prevention of vertical transmission from mother to foetus, as well as guidance and knowledge about various aspects of childbirth and newborn care [16]. PWLWHIV not only experience the usual doubts and anxieties of childbirth but also face challenges and uncertainties related to the virus itself [17]. Discrimination against these patients is still a major issue today [18,19].

The experiences of childbirth hold both immediate and long-term implications for a woman’s health and overall well-being. It can be a positive and empowering life event, mainly for PWLWHIV. PWLWHIV’s positive experiences of childbirth are often associated with expectations and have been found to have an impact on further treatment and their relationship with their child. Negative childbirth experiences have been associated with a greater likelihood of post-traumatic disorders, including postpartum depression. In addition, they can have a negative impact on the process of providing healthcare for the newborn and contribute to fostering fear of future childbirth experiences [20,21].

There is a significant body of evidence available regarding the childbirth experiences among pregnant women in general, antiretroviral therapy for Pregnant Women Living with HIV (PWLWHIV), as well as maternal–foetal vertical transmission of HIV. However, there remains a gap in understanding the specific childbirth experiences of PWLWHIV, encompassing the unique challenges and situations they encounter during this process. Therefore, there is a need to review the literature regarding the childbirth experiences of PWLWHIV. This evaluation aims to ascertain whether these experiences align with those of the broader pregnant population, identifying areas for potential improvement. Moreover, it seeks to explore any cultural and geographical variations that might exist in these experiences. Thus, a scoping review was developed, as it is the best indication to determine how much scientific literature covers a certain topic and give a clear indication of the volume of literature and studies available on this subject. It is very useful in examining emerging evidence that is not yet clear to map the existing knowledge on a specific theme or key question [22,23,24].

A scoping review was undertaken to explore the literature on the childbirth experiences of PWLWHIV. The objective was to uncover potential differences from the experiences of low-risk pregnant women and to determine whether positive childbirth experiences could enhance postpartum care, thereby facilitating the administration of antiretroviral drugs.

## 2. Methodology

A scoping review was developed, based on the Preferred Reporting Items for Systematic Reviews and Meta-Analyses (PRISMA guidelines) [25,26,27,28]. This review aims to map the available evidence on the childbirth experience of PWLWHIV from 2013 to 2023 (Supplementary S1, PRISMA Checklist).

Arksey and O’Malley [29] present a six-stage framework for conducting a scoping review, offering a systematic approach to literature search and a comprehensive foundation for guidance. However, in this particular study, the optional sixth stage involving consultation with practitioners and consumers was not employed, resulting in our framework comprising five stages. A protocol was established, involving two independent researchers who worked in a blinded manner to each other, aiming to minimize the potential for errors (DOI 10.17605/OSF.IO/WX52K) [29]. The research question was formulated using the Population, Concept and Context (PPC) strategy (Table 1), as recommended by the Joanna Briggs Institute Protocol (JBI) [23,24,27,30,31,32,33].

Stage 1: identify the research.

According to Arksey and O’Malley (2005) [34], stage one involves developing a question for the scoping review. To address this, the mnemonic PPC (Population, Concept and Context) was used. The population is pregnant women living with HIV (PWLWHIV). The concept is the personal experience or satisfaction of pregnant women during their own childbirth journey and the context of concurrently or subsequently caring for childbearing PWLWHIV.

The research question is as follows:

1. Is there evidence of the level of personal experience or satisfaction of PWHIV during their childbirth journey?

Stage 2: identify relevant studies.

The second stage of the framework is to identify relevant studies [34]. Following the definition of the research question, selection criteria were developed.

Inclusion criteria: studies involving PWLWHIV from 2013 to 2023 that evaluated their childbirth experience. No study was excluded based on the language of publication. All studies that investigated the childbirth experience of PWLWHIV, including both qualitative and quantitative studies, were included.

Exclusion criteria: studies that evaluated only mother-to-child transmission of HIV, the effectiveness of cARTS (combined antiretroviral drugs) or the evaluation of stigma and discrimination related to HIV infection. All studies conducted only during prenatal care were excluded. Editorials, reviews, case studies and opinion articles were excluded.

The search encompassed the following databases: MEDLINE, PUBMED, WEB of SCIENCE, Clinical trials org. The literature review was made by two independent researchers based on the following keywords: childbirth, experience, satisfaction, HIV, humaniz*, parturition, birth, perceived safety, maternal satisfaction, healthcare professional and midwi*.

**Figure 1:**
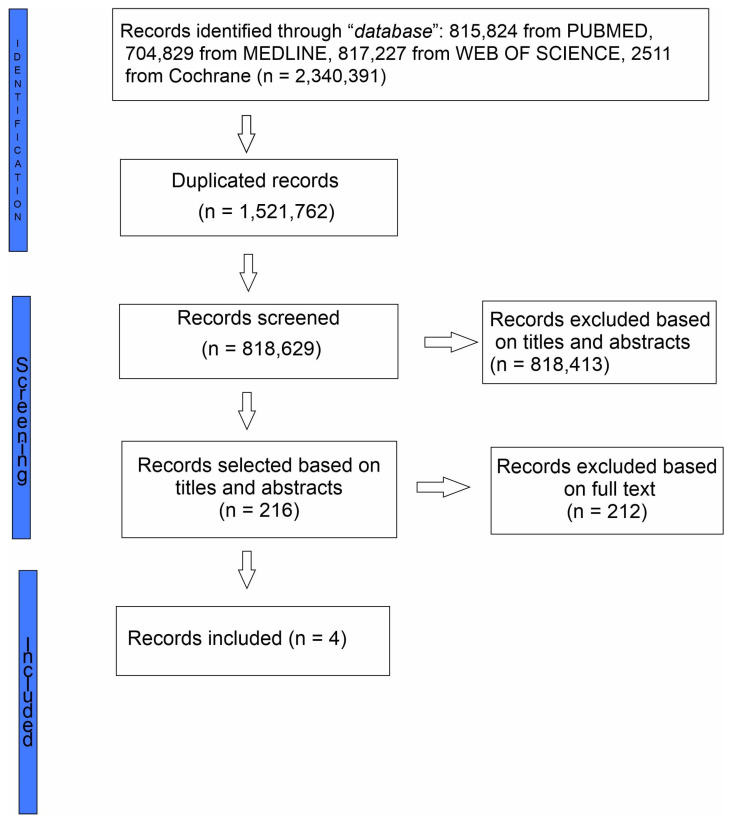
PRISMA flow diagram depicting the literature search methodology.

After the completion of work by the two researchers, a comparison was made regarding the articles selected by both researchers. In cases where discrepancies arose between their selections, a third researcher made the final decision regarding the inclusion of the particular article. The process of screening the data was carried out by reporting items for further systematic observation and potential inclusion in meta-analysis (PRISMA) (Figure 1) [25,28].

Stage 3: study selection

The study selection framework initially included 2,340,393 articles: 815,824 from PUBMED, 704,829 from SCOPUS/MEDLINE, 817,227 from Web of Science and 2511 from Cochrane. Among these, 1,521,762 duplicates were identified and subsequently removed. The 818,629 remaining studies underwent a review based on their titles and abstracts. Articles were included if the title indicated any information about PWLWHIV regarding childbirth or women’s personal experiences. Out of the initial pool, comprising 216 articles that underwent full-text review, upon closer examination, only 4 studies met the inclusion criteria for the scoping review (Supplementary S2).

A scoping review [34] addressing the experiences of PWLWHIV was identified. However, this particular review primarily focuses on the pregnancy experience, specifically emphasising prenatal and antenatal care. Consequently, it does not aim to explore the childbirth experience within its scope.

Stage 4: charting the data

The analysis of these 4 studies involved identifying the publication year, study type, characteristics and amount of the included population, examination of inclusion criteria, study location, variables encompassed in the study and the primary outcomes or main results.

Stage 5: collating, summarising and reporting

Each phase was extensively researched to ensure the broadest inclusion of studies. A search was also conducted using the keyword “HIV,” with the intention of encompassing all studies related to HIV, specifically seeking those that centred on the childbirth experiences of PWLWHIV. Most studies on childbirth aimed to evaluate vertical transmission between mother and foetus or antenatal/prenatal care.

## 3. Results

The characteristics of the included studies are displayed in Table 2.

All 4 studies [4,35,36,37] were carried out between 2014 and 2019. The studies were conducted in North America (Canada) (*n* = 2, 50%), Africa (Tanzania) (*n* = 1, 25%), and 1 in South America (Brazil) (*n* = 1, 25%).

Regarding methodology, all studies are original investigations: qualitative study (*n* = 3, 75%) and multicentric study of cohort (*n* = 1, 25%).

Regarding data collection instruments, the studies used interviews and questionnaires in most cases (*n* = 3, 75%), while one study (*n* = 1, 25%) incorporated direct observations of health professionals in addition to interviews (Table 1).

Regarding the selected population, 50% the studies were conducted exclusively with PWLWHIV (*n* = 2), while the remaining studies did not exclusively focus on them (*n* = 2, 50%). In one study, the diagnosis of HIV infection was confirmed years after the delivery occurred (Table 1).

When analysing the included studies, three main themes emerged: assessing the quality of the childbirth experience, vulnerability and autonomy.

### 3.1. Analysing the Quality of Childbirth Experiences

All four studies (100%) focused on exploring or analysing the experience of childbirth. Of these, the studies conducted exclusively with Pregnant Women Living with HIV (PWLWHIV) had a relatively small number of women included, with participant counts of 6 [35] and 66 [4], respectively. The remaining two studies included a larger number of pregnant women, including mothers without HIV infection [36,37]. However, these studies specifically analysed the importance of HIV status among the participants [36,37].

In three of these studies [4,35,37], only 30% to 40% of PWLWHIV reported having had a positive experience during labour. In contrast, in the other study, the majority of PWLWHIV (85%) rated their childbirth experience as positive [36]. Among the main complaints of PWLWHIV are the lack of involvement in the decision-making process regarding the method of delivery, insufficient guidance during prenatal care, minimal use of pain relief techniques and a lack of humanised care during childbirth [36].

### 3.2. Vulnerability

Of the two studies that examined the vulnerability of PWLWHIV [35,36], contrasting results were observed. Sando et al. [36] concluded that PWLWHIV did not show increased vulnerability compared to pregnant women without HIV infection. Bellotto et al. [35], on the other hand, found a different result. Their study indicated that PWLWHIV were more vulnerable, as they expressed greater concern about “saving the baby”—specifically, preventing vertical transmission of HIV—compared to prioritising their own childbirth experience. According to the author’s findings, a recurring fear of death or potential debilitation of the baby was observed, which suggests that the woman’s sense of guilt about having HIV was transferred onto the status of the child [35].

### 3.3. Autonomy of PWLWHIV

Of the studies analysed, only one [35] addressed the autonomy of PWLWHIV. Bellotto noted the insufficient attention given to the preferences and concerns of PWLWHIV regarding childbirth. The authors stressed that PWLWHIV were subjected to excessive medicalisation and suffered a loss of autonomy during childbirth. This lack of autonomy was exacerbated by the fear and guilt associated with the risk of vertical transmission, compounded by the emphasis placed on policies and health professionals [35].

## 4. Discussion

Pregnancy is an important time for testing and treating HIV [38]. The PWLWHIV’s experience during childbirth is critical for evaluating healthcare and the healthcare system, as well as maternity care [12]. Recognising and respecting the needs and desires of pregnant women are crucial in assessing maternity care and improving healthcare systems [39]. This is one of the goals of the 2030 Agenda for SDGs [1,2].

This review identified a gap in the literature regarding the evaluation of childbirth experiences of PWLWHIV. However, this subgroup is increasing every day. Initially, scientific studies focused on reducing the vertical transmission of HIV from mother to child. Now that this problem is minimised, we need to analyse the quality of childbirth experiences for PWLWHIV [28]. A positive childbirth experience may potentially enhance the adherence of PWLWHIV to the HIV care of their newborns.

This lack of quantitative studies on the childbirth experience of PWLWHIV was highlighted by Cichowitz [17,35], who specifically drew attention to the fact that studies prioritise the evaluation of vertical transmission of HIV and the retention of PWLWHIV in postnatal care and childcare services. Regarding these points, there are systematic reviews and meta-analyses in the literature. One systematic review draws attention to the fact that engagement in postnatal care depends on partner involvement, social support and childbirth experience [40]. Cichowitz [17] debates how the childbirth experience of PWLWHIV can influence adherence to the PMTCT in the postnatal period.

The four identified studies had small sample sizes, and none of them were thoroughly conducted with a specific focus on studying the childbirth experience of PWLWHIV. One study conducted by Fortin-Hughes et al. [37] analysed childbirths that had occurred up to 18 years before, and in these cases, the timing of HIV diagnosis was unknown. This lack of clarity regarding the HIV diagnosis timing might have introduced a notable bias in the recollection of the childbirth experience. Therefore, as the authors point out, despite the large number of women with HIV (905), the sample may not be representative of PWLWHIV, as many women did not have HIV at the time of childbirth [41].

Since the childbirth experience is difficult to measure, several questionnaires have been developed to assess all dimensions of this experience [10,42]. Blaszquez’s systematic review [12] on instruments used to measure patient satisfaction regarding childbirth experience included 17 high-quality studies in the literature, with the majority developed within Europe and related to healthy women with low-risk pregnancies. All questionnaires underwent reliability, content and validation aspects. According to this systematic review, the most widely used scale was the Mackey Childbirth Satisfaction Scale, which has been applied in the United States, Great Britain, the Netherlands, Belgium, Spain, Iran and Brazil [43,44,45,46,47,48,49,50]. Mackey’s Childbirth Satisfaction Rating Scale (MCSRS) questionnaire assesses two important dimensions of childbirth satisfaction: the first relates to the process of professional care (satisfaction with nurses, doctors, hospital and childbirth in general), and the other relates to personal satisfaction with the partner and family [43]. Those questionnaires gave a quantitative analysis to the qualitative studies that were found in this scoping review. Unfortunately, none of the four studies [4,35,36,37] used any questionnaire to outline women’s profiles in the various spheres of biopsychosocial and cultural contexts, such as the Labour and Delivery Satisfaction Index (LADSI) [51], Mackey Childbirth Satisfaction Rating Scale [43], Women’s View of Birth Labour Satisfaction Questionnaire (WOMBLSQ) [52], Perceived Control in Childbirth Scale (PCCh) [53], and Women’s delivery experience measures (MFRM) [54]. All four studies employed interview-based methodologies and exclusively conducted qualitative studies [4,35,36,37]. Measuring patient satisfaction is not easy. For a comparative analysis between women, institutions or the various facets of labour care, a quantitative measure is essential [53,55]. A satisfaction questionnaire (instrument) must consider a range of potential dimensions, including continuity of care, availability of carers, access, interpersonal skills and technical competence [55]. “Homemade” satisfaction questionnaires or just interviews tend to overestimate satisfaction, as do those which ask questions about satisfaction in general terms [52].

The narratives provided by PWLWHIV in these studies unveiled the settings where stigmatising practices arise while these women seek perinatal care and support. Additionally, these narratives shed light on the correlation between HIV-related stigma, disclosure and the consequential impact on women’s pregnancy and childbirth experiences [35]. Pregnant Women Living with HIV often experience mixed emotions: a sense of joy and fulfilment associated with maternity and childbirth, juxtaposed with feelings of fear, anxiety, and isolation [56]. PWLWHIV also experience stigma and discrimination when reaching the medical care system. On the other hand, it is revealed that, after knowing the treatment conditions (cART), they feel more secure and hopeful. It has been reported that through a multidisciplinary approach and emotional support during challenging periods, PWLWHIV rely on faith and the desire to live on, aiming to care for their children and witness their healthy growth and development. Health professionals play a crucial role in elucidating the distinctions between HIV infection and AIDS while also providing information about the effectiveness of combination antiretroviral therapy (cART) [57].

According to Hernandes et al. (2019), previously diagnosed PWLWHIV see pregnancy as an opportunity to overcome and materialise a correctly performed treatment, whereas PWLWHIV newly diagnosed during prenatal care may feel guilty, shaken and without the emotional structure to carry the pregnancy forward.

Childbirth care should follow general WHO recommendations. Any procedure that increases the baby’s contact with the mother’s blood, a break in the baby’s skin, such as scalp electrodes or suction cups, should be avoided due to the risk of increasing vertical transmission [58].

Like all healthcare consumers, PWLWHIV have the right to make autonomous decisions about their medical care. This includes the ability to decline to follow medical advice, guidelines or policy. In alignment with the guidelines of the World Health Organization [59], patients are entitled to several rights, including the right to information, informed consent or informed refusal and respect for their choices and preferences. They also have the right to confidentiality and privacy, to be treated with dignity and respect, to receive quality healthcare free from any form of violence and to be treated equally without discrimination. Additionally, patients have the right to receive the best and safe healthcare services and to exercise freedom, autonomy and self-determination, including the right not to be coerced.

The negative experiences of childbirth have become increasingly common nowadays. These negative experiences can cause postpartum psychological trauma (PBT) and lead to post-stress disorder (PTSD) [20,59]. Taheri et al. [60] carried out a systematic review of randomised clinical trials and a subsequent meta-analysis with the aim of identifying antenatal and intrapartum care practices that serve to prevent negative birth experiences. Taheri [61] evaluated 8685 studies between 1994 and 2016 and selected 20 studies for a meta-analysis in low-risk pregnancies. This systematic review divided the practices into four main groups to improve the childbirth experience: support actions during childbirth for the pregnant woman, pain relief and relaxation techniques during childbirth, intrapartum care with minimal intervention and childbirth preparation. Successful strategies included the presence of a trained birth companion, relaxation through massage and music, childbirth with minimal interventions and the development of an individualised birth plan. The utilization of these practices should be encouraged by maternal–foetal health programs, which are based on promoting vaginal birth, high-quality maternal care and reductions in chronic psychological complications [61]. The negative experiences of childbirth are associated with PBT and PTSD, disruption of interpersonal relationships, lack of care for the newborn and increased rates of caesarean sections in future pregnancies. In pregnant women living with HIV, this factor becomes increasingly crucial, as mothers are required to uphold their adherence to treatment in the postpartum period and take responsibility for the care of their newborns, including administering medications to them [60]. Insufficient neonatal follow-up can potentially increase infection rates among newborns [62,63,64,65,66]. Kreitchmann et al. demonstrated a significant decrease in adherence to combination antiretroviral therapy (cART) during the postpartum period in a multicentre cohort study conducted in Latin America [67].

The mistreatment and disrespect suffered by pregnant women during childbirth are increasing worldwide. A systematic review conducted by Bohren [62] evaluated qualitative and quantitative studies on the physical, sexual and verbal abuse suffered by pregnant women in general. For PWLWHIV, the issue of stigma and discrimination intensifies. A study conducted in Tanzania revealed that this mistreatment occurred in 14.8% of births [36].

Clouse et al. [60] studied 25 postpartum women between August and December 2016 (6 to 18 months postpartum period). Interviews were carried out to evaluate the continuity of child follow-up. The barriers identified included insufficient financial resources, disrespectful treatment by healthcare professionals, discrimination by healthcare providers, maternal lack of motivation and time constraints. This study underscored the crucial role of healthcare professionals and providers in ensuring a positive experience for any patient.

Like all healthcare consumers, PWLWHIV have the right to make autonomous decisions about their medical care. This encompasses the right to refuse to comply with medical advice, guidelines or policies. In accordance with the guidelines of the World Health Organization [63], the patient has the right to information, informed consent or informed refusal, and respect for their choices and preferences. They also have the right to confidentiality and privacy, to be treated with dignity and respect, to receive quality healthcare free from any form of violence and to be treated equally without discrimination. Additionally, patients have the right to receive the best and safest healthcare services and to exercise freedom, autonomy and self-determination, including the right not to be coerced [63,64].

The presence of a companion during childbirth is associated with a better childbirth experience for women [62,65,66]. In a study conducted in Ghana, Guinea and Nigeria, the absence of a companion is associated with higher rates of physical abuse, unauthorised and non-consensual interventions, lack of communication and respect [65]. The companion supports the pregnant woman in four distinct ways: providing emotional and physical support (including massage, verbal and physical encouragement, building trust), bridging the communication gap between healthcare professionals and the pregnant woman, facilitating pain relief through non-pharmacological alternative therapies (such as exercises, affection, music therapy) and advocating for the pregnant woman, safeguarding her privacy and preferences with healthcare professionals [62]. We did not come across any studies specifically addressing the presence of companions during childbirth for PWLWHIV.

From the perspective of the humanisation of childbirth care, non-pharmacological methods of pain relief can contribute to a positive childbirth experience. However, many hospitals do not have these methods available and/or do not have a proper work process and physical structure to employ them, either due to healthcare professionals’ lack of knowledge or the excess of medical interventions [35]. Unnecessary interventions, which the World Health Organisation considers unacceptable in humanised childbirth, have increased [63].

Qin et al. [68] analysed the psychological disorders of 194 PWLWHIV between June 2012 and August 2016 using anxiety and depression questionnaires, Berger’s HIV stigma scale or problem lists. The positive detection rate was 69.1%. The anxiety rate was 60.8%, and the depression rate was 54.1%. The demographic and social characteristics did not influence these rates. We excluded this study because it did not analyse the childbirth experience, despite examining the emotional aspects of pregnancy. However, it is important to show that PWLWHIV have more psychological problems than HIV-negative pregnant women [68].

A study in India showed that empowered women have a lower risk of negative childbirth experiences and obstetric violence [69]. Empowered women, driven by their self-assurance and awareness of their rights, assertively advocate for greater respect for their autonomy and preferences, reducing the likelihood of experiencing abuse and mistreatment within healthcare services. The fact is that we did not find any study on the positive or negative childbirth experiences of pregnant women living with HIV. Adverse experiences during childbirth can lead to psychological and emotional disturbances, potentially acting as barriers to adherence to postpartum treatment for women living with HIV and their children. It is essential that the health system prioritises respect for women, promotes and safeguards their dignity and, thus, increases their confidence [17].

## 5. Conclusions

The aim of this scoping review was to comprehensively explore and assess the quality of childbirth experiences among PWLWHIV and to identify the various factors associated with these experiences. It was identified as an area in the literature that is still lacking in terms of studies that need further research and clarification. Pregnancy in the presence of HIV does not necessarily mitigate the positive feelings of childbirth and motherhood, but it certainly instils fear and requires certain precautions to prevent vertical transmission and maintain a positive childbirth experience. It is a challenge for PWLWHIV and healthcare professionals to equally prioritise the prevention of vertical transmission of HIV and the humanisation of childbirth. The second major challenge is to reduce the negative childbirth experience, which has been increasing among pregnant women in general [70,71,72,73,74,75,76,77].

While recognising the need to adhere to prophylaxis protocols to prevent mother-to-child transmission and the essential medical interventions involved, it remains crucial to prioritise a humanised approach to childbirth for PWLWHIV. Empowering PWLWHIV to make decisions regarding birthing positions and delivery methods, as long as the viral load remains undetectable, is essential to promote a more humane birth experience. PWLWHIV must also have the choice of exploring non-pharmacological methods for pain relief. They can also have the presence of a companion during the entire childbirth process [78,79].

For our society, according to the Universal Declaration of Bioethics and Human Rights [80], the autonomy of PWLWHIV should always be guaranteed and encouraged [81,82,83].

## 6. Areas for Further Research

Further research is essential to evaluate the potential impact of comprehensive prenatal care and childbirth education in mitigating the fears experienced by women living with HIV during pregnancy and delivery. Additionally, emphasizing informed rights during prenatal care may aid in fostering positive childbirth experiences. Addressing this informational aspect during prenatal care could potentially reduce the discontinuity between prenatal care and childbirth [35]. Moreover, continued education for healthcare professionals is crucial to increase the involvement of PWLWHIV in decisions regarding their childbirth experiences [84,85,86].

Conducting additional studies to assess the satisfaction levels of PWLWHIV regarding their childbirth experiences is imperative, using a quantitative and validated data collection instrument, such as the Labour and Delivery Satisfaction Index (LADSI) [51], Mackey Childbirth Satisfaction Rating Scale [43], Women’s View of Birth Labour Satisfaction Questionnaire (WOMBLSQ) [52], Perceived Control in Childbirth Scale (PCCh) [53], and Women’s delivery experience measures (MFRM) [54].

## Figures and Tables

**Table 1:** PPC.

P-population	Pregnant women living with HIV
C-concept	Satisfaction or experience of pregnant women at childbirth
C-context	Childbirth

**Table 2:** Characteristics of the included studies (*n* = 4).

No	Title/Author/Year	Country	Aim	Type of Research	Data Collection	Sample Size	Results
1	Between the Woman and saving the baby: HIV positive women’s experience of giving birth (Bellotto et al., 2019 [35])	Brazil	To analyse the experience of childbirth of women living with HIV.	Qualitative approach and psychological analysis	Many in-depth interviews for each woman living with HIV	6 women living with HIV	Women living with HIV expressed greater concern about ensuring the health of their newborn, preventing transmission of HIV, rather than focusing on their own childbirth experience. Only two women who had previously undergone childbirth were concerned about their own experience and autonomy during the process.
2	“Why are you pregnant? What were you thinking?: How women navigate experiences of HIV-related stigma in medical settings during pregnancy and birth. (Greene at al., 2016 [4])	Canada	To understand and respond to women’s unique experiences and psychological challenges during pregnancy and birth.	Qualitative study	Narrative interviews	66 pregnant women living with HIV	The narratives of women living with HIV reveal the environments where stigmatising practices arise as these women seek perinatal care and support. Additionally, these narratives shed light on the correlation between HIV-related stigma, disclosure, and their profound impact on women’s pregnancy and childbirth experiences.
3	Disrespect and abuse during childbirth in Tanzania: Are women living with HIV more vulnerable? (Sando et al., 2014 [36])	Tanzania	To explore instances of disrespect and abuse during childbirth in Tanzania, the study aimed to compare experiences between pregnant women living with HIV and those not living with HIV.	Prospective qualitative study (interview)	Mixed-method design post-partum interviews, direct observation (208), in-depth interviews (18), health care provider self-report (50)	HIV + 147 (7%) HIV − 1807 (91%) 2% unknown HIV status	Among women living with HIV and those who are HIV negative, 12.2% and 15.0% respectively reported experiencing disrespect and abuse during childbirth (*p* = 0.37). No significant differences were found between the experiences of women living with HIV and HIV-negative women in various forms of disrespect and abuse, except for women living with HIV, who exhibited higher odds of reporting non-consented care (*p* = 0.03).
4	Previous experiences of pregnancy and early motherhood among women living with HIV: a latent class analysis. (Fortin-Hughes et al., 2019 [37])	Canada	To analyse how previous childbirth experiences influence the current childbirth experience.	Multicentric study in Canada Cohort Study	Survey/questionary	905 women living with HIV	The analysis revealed that the majority (70.8%) of pregnancies occurred before the HIV diagnosis. A four-class maternity experience model was identified, comprising the following categories: “overall positive experience” (40%), “positive experience with postpartum challenges” (23%), “overall mixed experience” (14%), and “overall negative experience” (23%). Furthermore, no correlations were found between the timing of HIV diagnosis (before, during, or after pregnancy) and the identified patterns of childbirth experience.

## Supplementary Materials

The following supporting information can be downloaded at: https://www.mdpi.com/article/10.3390/children11060743/s1, Supplementary S1: PRISMA Checklist; Supplementary S2: Screening Database. Reference [87] is cited in the Supplementary Materials.
